# Engagement in e-cycling and the self-management of type 2 diabetes: a qualitative study in primary care

**DOI:** 10.3399/bjgpopen18X101638

**Published:** 2019-04-17

**Authors:** Aidan Searle, Emma Ranger, Jez Zahra, Byron Tibbitts, Angie Page, Ashley Cooper

**Affiliations:** 1 Senior Research Associate, BRC Nutrition Theme School of Oral and Dental Science, University of Bristol Education and Research Centre, Bristol, UK; 2 PhD Student, Centre for Exercise, Nutrition and Health Sciences School for Policy Studies, University of Bristol, Bristol, UK; 3 Senior Research Associate, BRC Nutrition Theme School of Oral and Dental Science, University of Bristol Education and Research Centre, Bristol, UK; 4 Senior Research Associate, Centre for Exercise, Nutrition and Health Sciences School for Policy Studies, University of Bristol, Bristol, UK; 5 Professor of Physical Activity and Public Health, Centre for Exercise, Nutrition and Health Sciences School for Policy Studies, University of Bristol, Bristol, UK; 6 Professor of Physical Activity and Public Health, Centre for Exercise, Nutrition and Health Sciences School for Policy Studies, University of Bristol, Bristol, UK

**Keywords:** E-cycling, exercise, Primary Care, Self-Management, Qualitative, diabetes mellitus, type 2

## Abstract

**Background:**

Physical activity (PA) is important in the management of type 2 diabetes (T2DM), however many people find it difficult to implement and/or sustain in the self-management of the condition. Electrically assisted cycling (e-cycling) may be viewed as a means of self-management in which effort is invested to balance the interplay of lifestyle factors and disease progression.

**Aim:**

To explore engagement with an e-cycling intervention conducted with adults with T2DM.

**Design & setting:**

Prospective qualitative interview study with adults in central Bristol (UK) and surrounding suburbs, in the context of the self-management of T2DM in primary care.

**Method:**

Interviews were conducted with 20 individuals with T2DM (42–70 years, 11 male, 9 female) prior to their participation in a 20-week e-cycling intervention. Post-intervention interviews were conducted with 18 participants (11 male, 7 female). Interviews were transcribed verbatim and inductive thematic analysis was undertaken.

**Results:**

Participants were aware that PA contributed to the management of their diabetes. Engagement with e-cycling was viewed as both an acceptable and a social lifestyle intervention. Furthermore, participants were unhappy with the volume of medication used to manage their diabetes and e-cycling fostered autonomy in the management of T2DM. GPs and practice nurses were regarded as an important source of reliable information, and were considered to be best placed to talk about interventions to increase PA.

**Conclusion:**

E-cycling is viewed as an acceptable form of PA to aid the self-management of T2DM. E-cycling may support people with T2DM to reduce their medication intake and in turn foster greater autonomy in managing the condition. The findings have implications for the role of primary care health professionals in supporting both patients and significant others in adoption of e-cycling.

## How this fits in

This prospective qualitative study highlights how e-cycling is an acceptable form of PA that may help people with T2DM to reduce their medication and foster self-management of the condition. Importantly, the study has highlighted the role of primary care health professionals in the provision of support for both patients and significant others in the adoption of e-cycling.

## Introduction

PA is important in managing T2DM, however many people find it difficult to implement and/or sustain such lifestyle changes following diagnosis.^1^ Furthermore, behavioural interventions in people with T2DM have had variable success in initiating and sustaining PA at levels that produce clinically meaningful health improvements.^2–4^ Consequently, there is a need to investigate the effectiveness of interventions designed to produce long-term sustainable changes in PA levels, which facilitate behaviour change beyond the intervention period.^5^

The adoption of ‘active travel’, such as walking and cycling, represents a convenient way of fostering engagement in daily PA. It was found, for example, that active commuting was associated with levels of moderate to vigorous PA in men and women with T2DM when compared to those commuting using motorised transport.^6^ With half of all car journeys in the UK being 1–5 miles in length, the substitution of many car journeys for walking and/or cycling may be an achievable means of engagement in PA.^7^

The sustainable and wide-reaching benefits of cycling support the case for promoting cycling as a potential tool in improving public health.^8^ However, rates of cycling are low and common barriers to cycle-commuting include the physical constraints associated with hilly terrain, poor physical fitness, lack of time, and the distance to work.^9^ Electrically-assisted bicycles (e-bikes) can help individuals to overcome some of these perceived barriers.^10^ E-bikes provide electrical assistance only when the rider is pedalling (through sensors which detect pedalling speed and force) and can help to motivate novice cyclists, and increase the likelihood that these individuals will continue to cycle in the future.^9^ A recent systematic review has shown that although e-cycling is less intense than conventional cycling, e-cycling was predominantly conducted at moderate to vigorous intensity (an intensity of >3 metabolic equivalents [METs], which is energy expenditure three times greater than resting).^11^ Thus, e-cycling could contribute to meeting PA recommendations for health (≥150 minutes of at least moderate intensity activity each week).

PA is a fundamental aspect of diabetes care, and the development of self-management is an important component of this.^12^ Self-management of illness can be defined as a '*process in which individuals acquire skills, strategies, and knowledge to manage the physical, psychological, emotional, and social effects of a chronic condition’.* ^13^ However, professional support for patients with T2DM may be lacking, such that *‘there is little or no prospect of achieving an intended outcome’.* Indeed, many patients with chronic disease that could be self-managed do not have the coping skills and/or are supported in their attempts at self-management and implementation of lifestyle change.^14^

E-cycling may be viewed as a means of self-management in which effort is invested to balance the interplay of lifestyle factors and disease progression. The use of medication for glucose and blood pressure control are central to the management of T2DM.^12^ While effective, such treatment can involve side effects and may make patients feel that they lack control or influence in the progress of their disease. It is proposed that e-cycling can help to initiate and sustain PA, and may assist in the self-management of T2DM. This qualitative study explores participants’ views of engagement with an e-cycling intervention in the context of the self-management of T2DM.

## Method

### Participants and recruitment

Potential participants for the study (Promoting Electrically-assisted cycling in people with type 2 Diabetes: Acceptability and feasibiLity [PEDAL]) were identified via an existing database of 99 people newly diagnosed with T2DM (HbA_1c_ >48 mmol/mol [>6.5%]) who had participated in the STAMP-2 study (Sedentary Time and Metabolic Health in People with Type 2 diabetes), a cross-sectional, observational study of ‘usual’ sedentary behaviour in adults with, or at high risk of, T2DM. All participants in STAMP-2 had previously agreed to be contacted regarding future studies and were informed of the proposal to conduct an e-cycling project.

### Patient–public involvement

To explore initial perceptions of an intervention utilising e-bikes, a patient–public involvement (PPI) meeting was held in June 2015 prior to designing the PEDAL study. Individuals who expressed an interest were invited to attend a meeting with members of the research team and representatives from a third sector cycle charity (Life Cycle UK) to discuss ideas about using e-bikes in the context of managing T2DM.

A presentation about the proposed project was given and questions from the potential participants were taken. An example of an e-bike was demonstrated in the meeting during which people had the opportunity to try the bike and converse with the research team. Opinions from participants at this meeting were used to shape the PEDAL intervention, and subsequent PPI meetings served to inform the design of the study and content of materials. Findings from the study have been provided to participants through subsequent PPI meetings, a newsletter, and a video web-link (https://www.youtube.com/watch?v=-rAp15If1Uk.).

Recruitment to the PEDAL study was conducted from May–June 2016. Responders who had returned a postal expression of interest form were contacted by the PEDAL project coordinator via telephone or email, and sent a participant information sheet. Participants were also given a verbal explanation of the study and the opportunity to ask questions prior to consenting to participate.

Twenty-eight of 99 people from STAMP-2 expressed an interest in participating. Of these, 20 eventually agreed to participate. Twenty participants took part in the interviews between May–October 2016, comprising 11 men and nine women aged between 42–70 years (mean 58 years). Nine participants were either retired or not working. Although all participants declared themselves cycling proficient, only 14 owned a bicycle at the time of study recruitment (Table 1).

**Table 1.** Pre-intervention demographic data (*n* = 20)

	Men, *n* (%) (*n* = 11)	Women, *n* (%) (*n* = 9)	Total, *n* (%) (*n* = 20)
Mean age, years (SD)	57.5 (9.31)	58.7 (5.20)	58.0 (7.64)
Ethnicity (% white)	91	100	94.7
Working	6 (54.5)	4 (44.4)	10 (50.0)
Not working/retired	5 (45.5)	4 (44.4)	9 (45.0)
Bike owner	9 (81.8)	5 (55.6)	14 (70.0)
Cycle proficient	11 (100.0)	8 (88.9)	19 (95.0)

SD = standard deviation.

### Design

The PEDAL study was a feasibility study aimed to: determine whether e-cycling was acceptable to people with T2DM; determine whether an e-bike would be used if provided; explore outcome measures for future studies; and describe the experience of using an e-bike to inform intervention development. The details of this study have been reported elsewhere.^15^ Briefly, participants' height and weight were measured, and their clinical history taken. After baseline measurements, participants individually met a cycle instructor from Life Cycle UK to familiarise them with the e-bike and to provide cycle training on local roads, including guidance on safe riding practices. Participants were then provided with an e-bike, helmet, gloves, reflective bib, lock, and panniers to use for 20 weeks. Support was provided for any mechanical problems encountered by participants. The built-in e-bike odometer values were recorded at the start and end of the loan period to measure total distance cycled.

### Interviews

Semi-structured interviews were conducted with participants in the PEDAL study. These interviews were conducted at two time-points: pre-intervention and post-intervention. Interviews were conducted by two experienced qualitative researchers and a PhD student with qualitative experience. For continuity, each interviewer conducted follow-up interviews with the same participants they interviewed pre-intervention.

Interviews were conducted with all 20 participants before the one-to-one cycle training. A topic guide was developed to inform the semi-structured interviews. The guide was discussed and reviewed by the wider PEDAL research team before the final version was agreed. The guide included items relating to employment and lifestyle, reasons for participation, perception of T2DM, attitudes toward PA, past cycling experience, and motivation to cycle.

### Follow-up

Following the 20-week intervention, interviews were conducted with 18 of the participants who had been interviewed pre-intervention. One participant withdrew following baseline measures and one participant was unavailable for interview. These interviews were based on a follow-up topic guide that included experience and day-to-day practicalities of using the e-bike; decisions to e-cycle; cycle training; barriers to e-cycling; perception of T2DM; changes in travel modes; motivation; and future recommendations for study recruitment and execution.

### Analysis

Data collection and analysis were undertaken concurrently; interviews were transcribed verbatim and reviewed. In keeping with the iterative process of qualitative research, modifications were made to the topic guide based on data from initial interviews. For example, initial interviews suggested that motivation to e-cycle was associated with significant others also participating, thus an item to capture social interaction was introduced as a topic.

Thematic analysis was then undertaken following the guidelines stipulated by Braun and Clarke.^16^ Inductive thematic analysis is described as a flexible and useful research tool for psychological research that provides a rich, detailed, and complex account of data.^17^

First, the analysts read and re-read the transcripts to familiarise themselves with the dataset. Second, initial codes were identified in a sub-sample of four interviews. When comparing codes, differences were discussed thoroughly until consensus was achieved, and a coding frame was developed. Third, the coding framework was independently applied to a further three interview transcripts by the analysts. Each section of coded text was reviewed to ensure consistency, and where discrepancies were identified, they were discussed until consensus was achieved and the coding frame was revised accordingly. High consensus between coders suggested that the codes sufficiently covered the data, and coding of the remaining transcripts proceeded. During this coding, any new data that were not covered by the initial framework was discussed between analysts and, if appropriate, a new code was developed. Fourth, once all the transcripts had been coded, emerging themes were discussed. The themes were reviewed and discrepancies were discussed until consensus was achieved. These themes are presented in the results section with illustrative quotes from interviews with participants.

## Results

### Lifestyle and attitudes towards PA

Participants’ identified their lifestyle as a cause of T2DM, and lifestyle was generally perceived as a greater contributor to development of the disease than hereditary factors:

*'Probably, like I said, there is no history in my side, but then my own lifestyle has probably contributed a lot to … Food-wise. I don't eat like I should. I know what I've got to do.'* (P101, F, 58 years, pre-intervention)

However, some participants expressed resignation towards developing T2DM if it was present in their family, and they were not prepared to be too restrictive in their lifestyle choices and management of T2DM:

*'I am sure it partly is my lifestyle because sometimes I drink a bit too much. Like I say, my mum was a PE* [physical education] *teacher, how much more active can you get than that? She got it and part of me just thinks “be sensible, be careful”, but at the end of the day what will be will be. I believe it was always going to happen just from looking at my cousins. It happened to them, so there is no reason why it wouldn’t happen to me. I just try to be sensible with my diabetes. I am not going to live like a saint just because I have got it.'* (P116, M, 42 years, pre-intervention)

Participants were also disappointed that their past level of activity was not sufficient to have prevented them from developing T2DM:

*'It is recognised that exercise helps and is beneficial and can ward off so many things. You don’t go a day without hearing about one medical condition or another, if not ten, that can be improved or avoided by exercise. The fact that even before I stepped up things I thought I was doing a reasonable amount of exercise. I suppose I was a bit annoyed that that hadn’t helped ward it off.'* (P117, M, 65 years, pre-intervention)

### Self-management of T2DM and e-cycling

Most participants recognised that T2DM could lead to serious health complications if it was not well managed, and this was a motivator for engagement with PA and other lifestyle changes:

*'I think if I don’t do it now* [physical activity] *then I’ll suffer the consequences later. I hear horror stories about diabetes and what it can do to the body. So, I think if I don’t do it now and strike while the iron’s hot then I’m just asking for trouble later.'* (P102, F, 58 years, pre-intervention)

Furthermore, post-intervention, most participants believed that T2DM can be managed effectively through lifestyle choices rather than medication:

*'I think, actually, the message is, there is two kinds of message out there with diabetes, in terms of, “Once you’ve got it, that’s it, you’ve got it, right? It’s a long-term disease. You get free prescriptions for the rest of your life. That’s it.” And there’s another message out there that says, “Actually, if you go on a really intensive diet for eight weeks, increase your exercise levels, bring down all these key factors, you can get rid of it, and it will disappear.'* (P110, M, 54 years, post-intervention)

Some participants expressed an opinion that PA could potentially serve in reducing their blood sugar levels and were disappointed in not being able to lower their reliance on medication following the intervention

*'I would like to reduce some of the medication I am on because I am on some quite high doses of some stuff. It is still early days, I am only two years into having the diabetes and they are still tinkering around with my medication to try and get everything balanced. I definitely think exercise is an important part of it. You have to take a bit of medication. I think exercise can naturally bring down your sugar levels, which is why I was a bit disappointed that not a lot had changed.'* (P116, M, 42 years, post-intervention)

*'I’m on a very small dosage of drug for high blood pressure. He just monitored my blood pressure, and it was 110 over 80, something or other. So maybe I don’t need to be on that. And that’s a discussion I’m going to have with my doctor. “Look, if I keep up this level of exercise, can I come off that?” Because the more drugs I can come off, the better.'* (P110, M, 54 years, post-intervention)

E-cycling served some participants by increasing their autonomy in the management of T2DM and they suggested that they would continue to cycle in the future:

*'Well, I was going to the gym and I find the gym so dead boring, but that was having quite a good effect on my diabetes, but it started to peter out. I thought, “The reason it’s petering out is because I’m in a gym and I really don’t like being in that atmosphere. I’d much rather be outside.” I used to play a lot of tennis, but it’s not easy to find people to play tennis with. Whereas, a bike, you can just go … It’s like running, you can just go and suit yourself. So, I thought that for me was worth a lot.'* (P102, F, 58 years, post-intervention)

### Perceived benefit of e-cycling

Some participants positively attributed improvement in blood glucose levels and weight loss to engagement with e-cycling:

*'I think the electric bike’s down to starting exercise, and the benefits of exercise relating to the diabetes. And there has been an improvement in the diabetes. Six-monthly review was fine. But actually, sort of, the average blood sugar level was back down below the trigger point for the diabetes. The weight is coming off. I’ve lost another stone in the process of doing this. Bearing in mind that I haven’t changed anything else.'* (P118, M, 52 years, post-intervention)

When asked if the e-cycling intervention had served participants in their intention to integrate other forms of PA into daily life, many expressed it had raised levels of motivation for activity:

*'I’m still very motivated … I just need to make sure I can still get what I call a lot more informal exercise, when you’re just kicking a ball around a field and things like that with my sons … With having a lot more things to do and other things, commitments and things like that. I want to maintain that definitely, doing lots of walks things like that. I think those are the ways to get you exercising a lot. Some people don’t enjoy going to the gym and I’m probably one of those so you need to sort of trick yourself into getting exercise by doing something you enjoy that is also physically good.'* (P105, M, 44 years, post-intervention)

E-cycling also provided participants an opportunity for social interaction by riding with friends and significant others who may have had more cycling experience and/or stamina:

*'Yes, we managed to get out quite a lot during the summer, sort of like on the weekends more than anything else. I didn’t use it a lot during the week, but on the weekends we, sort of, did the two tunnels and went then to* [location]*, and it was fun, sort of like, leaving him behind, sort of going up hills and that. We’d go out for meals as well, which was really good, because I was able to cycle a lot further than normal*.' (P107, F, 49 years, post-intervention)

### Future recommendations for studies and primary care

Many participants felt that communication via primary care would be the most appropriate and accessible way to disseminate information regarding opportunities to participate in interventions:

*'I would’ve thought it’s got to go through the doctor’s surgery or something like that, with flyer things that the diabetic nurse could give them and say, “Would you be interested in this? If so, please sign this bit of paper.” Knowing now, I think I’ve probably done the right thing in doing it. If somebody had come up and said to me before, just off the cuff, “Do you want to go on a push bike?” “No.” But because of being diabetic and thinking about it a bit stronger, it’s helped.'* (P103, M, 67 years, post-intervention)

GPs and practice nurses were regarded as an important source of reliable information and considered to be best placed to talk about interventions to increase PA:

*'So, if that doctor, or nurse practitioner, or whoever it is, is saying, “Okay, so I can give you these tablets, but have you thought about more exercise?” And, “Oh, by the way, there’s this study,” and really sold the benefits of it, then I think that’s more likely to be a route that they will take it up.'* (P110, M, 54 years, post-intervention)

## Discussion

### Summary

Engagement with an e-cycling intervention was explored in the context of the self-management of T2DM both pre- and post-intervention. Participants conveyed their views with regard to the role of PA in the self-management of T2DM pre-intervention, and their engagement in the e-cycling intervention appeared to foster autonomy post-intervention. The present findings suggest that e-cycling is an acceptable form of PA for individuals with T2DM that could potentially be supported in the context of primary care.

### Strengths and limitations

A strength of the study was the use of a novel PA intervention that was acceptable in terms of use for different contexts of PA: commuting, recreational, and utilitarian.^15^ The study was also novel in sampling individuals newly diagnosed with T2DM and interviewing them both pre- and post-intervention. However, both the primary interviewers and data analysts were commuting cyclists at the time of the study and may have unwittingly influenced participants’ responses in the interviews and interpretation of the data. However, having a third data analyst who was not a cyclist may have reduced potential for bias in the findings. The hilly topography and traffic congestion in the study location may have impacted on the uptake of the e-cycling intervention. Finally, the ‘self-selected’ participants may not be representative of the wider population of the study from which they were recruited (STAMP 2), or of the general population of people with T2DM.

### Comparison with existing literature

Research in the context of epilepsy has shown that individuals may employ strategies of independence in self-managing their condition that include reflections on personal resilience as well as actual behavioural strategies, such as problem solving and self-care activities.^17,18^ Studies have also shown that these automated and unconscious habits override any decision-making or choice, and influence travel behaviour over and above attitudes and intentions.^19^ Others assert that only high ‘habit strength’ may have this effect,^20^ and that social and personal norms remain important.^21^ However, there was little evidence in the present data to suggest that habituation to e-cycling had occurred.

The findings suggest a role for significant others in the maintenance of e-cycling, and highlight the importance of contextual factors in self-management behaviour.^13^ For example, decisions to e-cycle in the context of the active travel intervention were often cued and supported by the opportunity for social interaction while cycling, which is consistent with social norms theory.^21^ How patients behave and cope with a chronic condition is mediated by contextual factors, particularly one’s social network and collaborative partnerships with health professionals, from whom they may seek support^13^ and who may serve to initiate and sustain behaviour change.

Previous qualitative research has shown that significant others and health professional–patient relationships can enhance perceptions of competence and autonomy in the context of behaviour change,^22^ which resonates with the construct of self-efficacy.^23^ Participants’ decisions to e-cycle were perceived to be within their control and this, in turn, fostered self-efficacy beliefs that sustained their capacity to e-cycle.

A recent review calls for a holistic model of behaviour change theory in the specific context of guiding active travel research.^24^ Included in this review is a synthesis of behaviour and psychological theories which make a distinction between reasoned influences on behaviour — such as perceptions, preferences, and attitudes — and unreasoned influences driven by habits and impulsiveness. Behaviour in this framework is depicted by a set of levels that range from the most short-term travel behaviour to integration into lifestyle. It is also posited that individuals’ behaviour is determined by opportunities and constraints which present themselves at the individual level, as well as through the social and spatial environment.

### Implications for research and practice

The e-cycling intervention was viewed as an acceptable means of adopting PA in the self-management of T2DM. Indeed, health professionals may be well placed to challenge individuals’ perceptions of PA in order to initiate and sustain e-cycling. However, there is a need for further research if the long-term health impact and financial implications of promoting e-cycling in the context of T2DM is to be evaluated. Furthermore, the viability of accessing e-bikes is subject to individual resources, and health professional support may increase costs in primary care. However, potential reductions in medication through adoption of PA, and the possibility of deferring or preventing future complications, may substantially offset or outweigh such costs.
